# Art of imaging: brain sagging in spinal cerebrospinal fluid leak

**DOI:** 10.1093/radadv/umaf041

**Published:** 2026-03-06

**Authors:** Parnian Habibi

**Affiliations:** Neuroradiology Research Fellow, Mayo Clinic, Rochester, MN 55905, United States

**Keywords:** spinal CSF leak, spontaneous intracranial hypotension (SIH), CSF-venous fistula (CVF)

**Figure:**
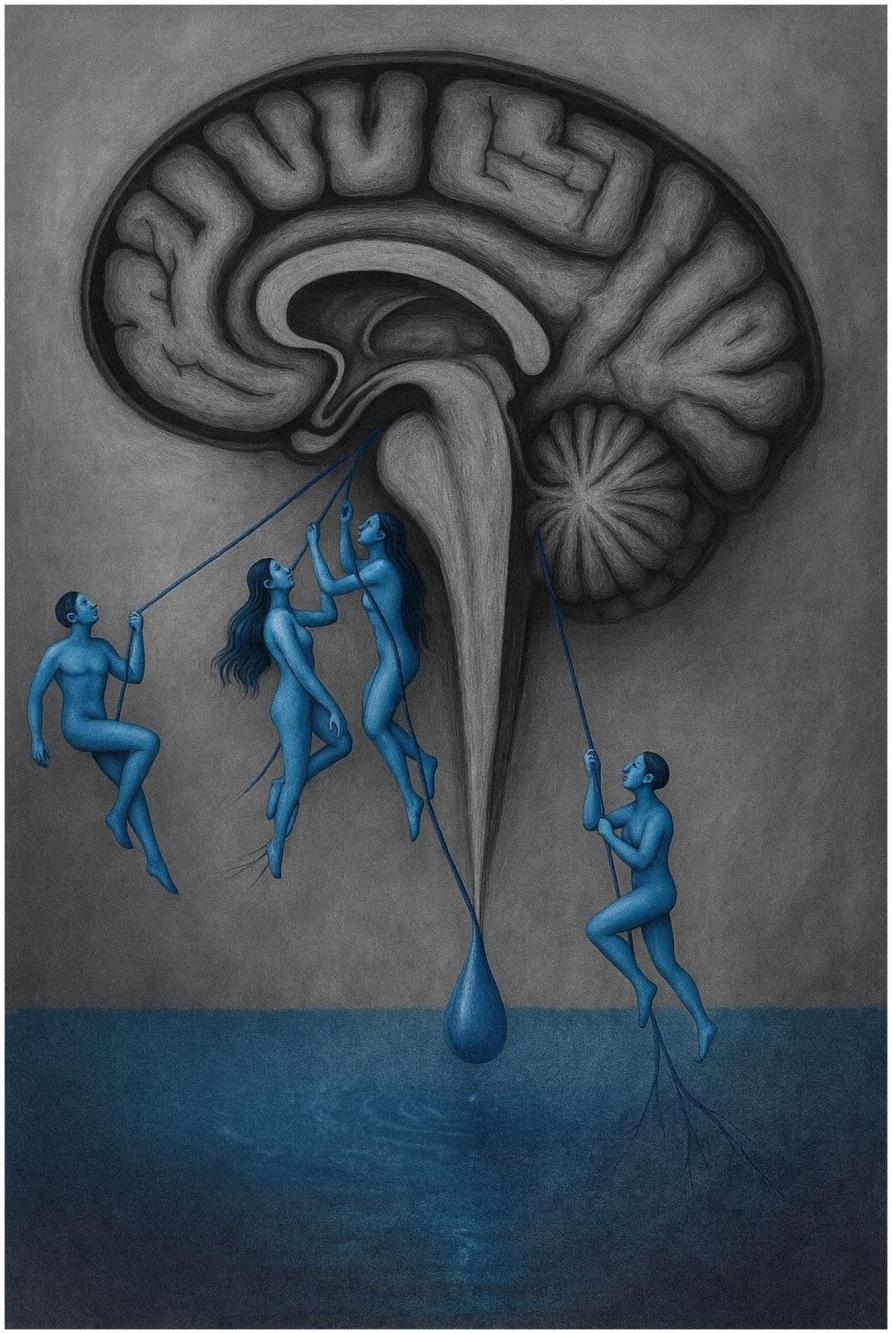
Human-like forms of leaking cerebrospinal fluid pull the brain downward, reflecting brain sagging and tonsillar herniation on MRI.

**Figure:**
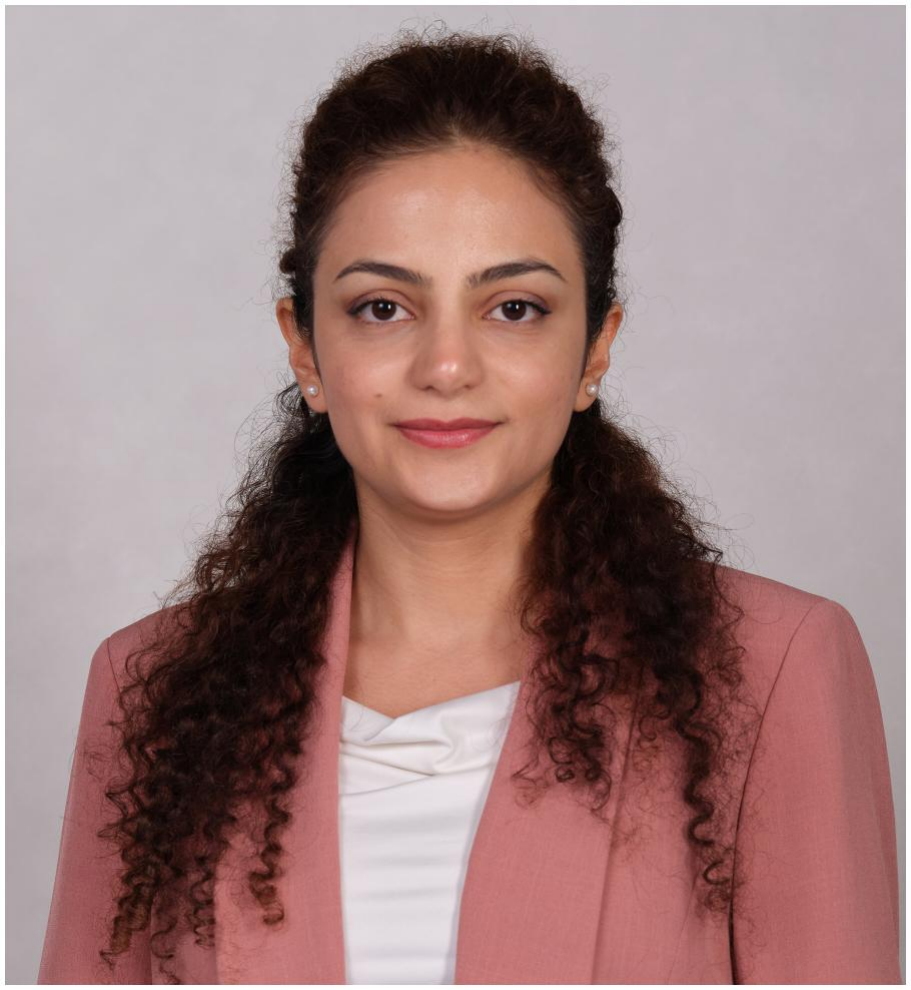
Parnian Habibi, MD Neuroradiology Research Fellow, Mayo Clinic, Rochester, MN, United States

## Supplementary material

Supplementary material is available at *Radiology Advances* online.

## Funding

None declared.

## Conflicts of interest

Please see ICMJE form(s) for author conflicts of interest. These have been provided as supplementary materials.

## Supplementary Material

umaf041_Supplementary_Data

