# Supplementary material for: Art of imaging: brain sagging in spinal cerebrospinal fluid leak
Source: Radiol Adv. 2026 Mar 6;3(2):umaf041. doi: 10.1093/radadv/umaf041 (PMC12965401; doi:10.1093/radadv/umaf041)
Supplement: umaf041_Supplementary_Data [file umaf041_supplementary_data.docx]

**Title: Art of Imaging: Brain Sagging in Spinal CSF Leak**

**Figure 1.**

[See uploaded Figure 1 file]

Caption: Human-like forms of leaking CSF pull the brain downward, reflecting MRI-based sagging and tonsillar herniation.

**Author Photo.**

[See uploaded Photo 1]

Biographic Information

Parnian Habibi, M.D., Neuroradiology Research Fellow, Mayo Clinic, Rochester, MN, USA
